# Supplementary material for: Comparative Analysis of Single-Molecule Dynamics of TRPV1 and TRPV4 Channels in Living Cells
Source: Int J Mol Sci. 2021 Aug 6;22(16):8473. doi: 10.3390/ijms22168473 (PMC8395219; doi:10.3390/ijms22168473)
Supplement: Supplementary file 1 [file ijms-22-08473-s001.zip › SupplementaryFiles/MDPI_SupplementaryFigure_210805v.pdf]

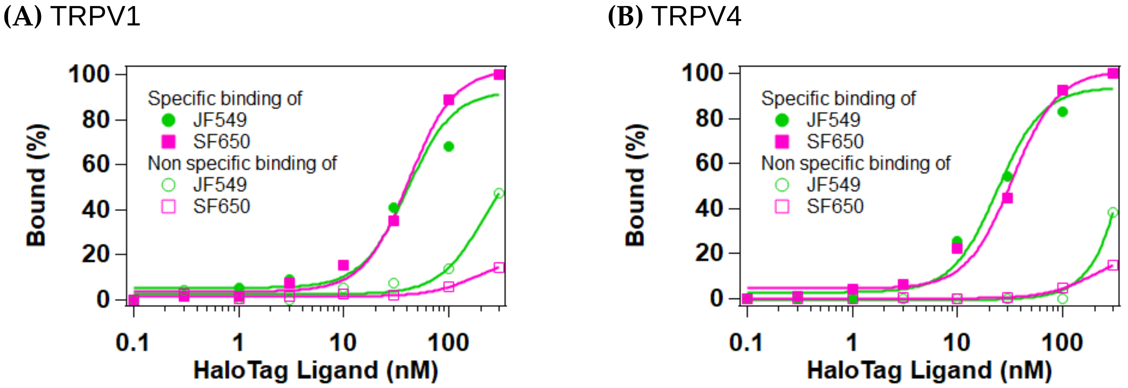

Figure S1. Saturation binding assay. (**A** and **B**) Specific and nonspecific binding of the JF549 and SF650 ligands to TRPV4-KO HEK293 cells expressing HaloTag-fused hTRPV1 (**A**) or HaloTag-fused hTRPV4 (**B**). Data are means of experiments performed in duplicate.

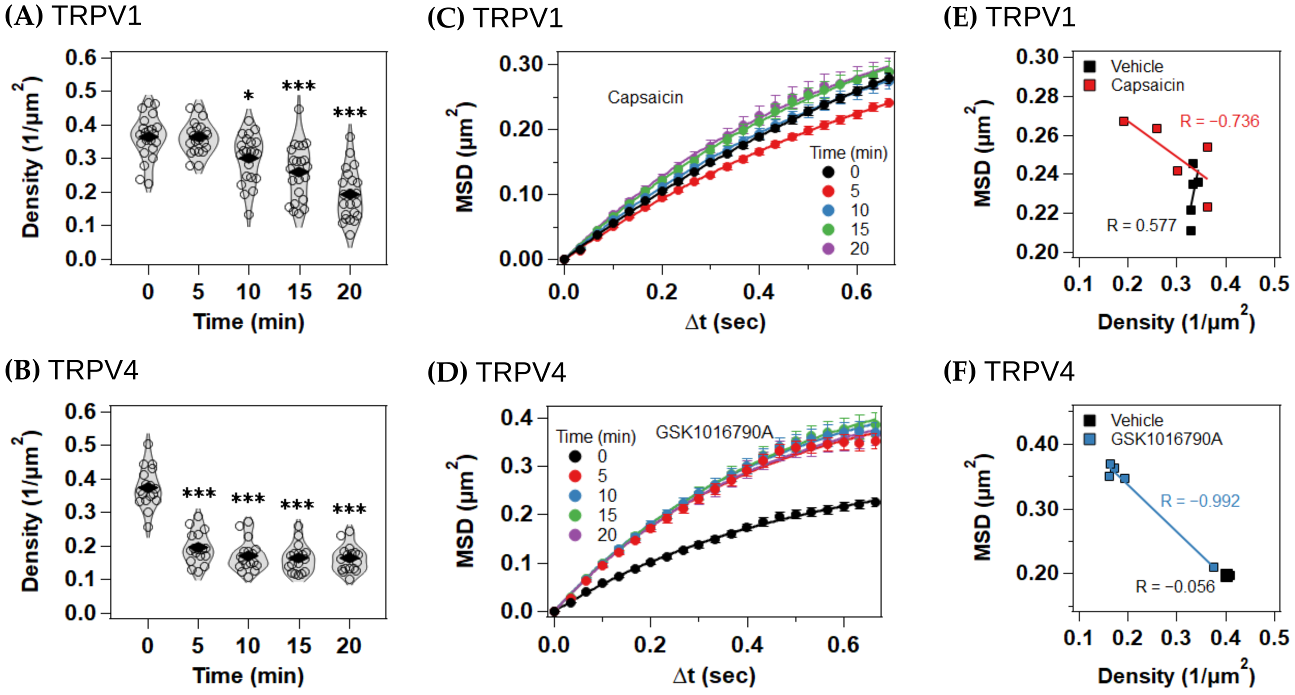

Figure S2. Receptor density and MSD–Δt plots of TRPV1 and TRPV4 channels after activation. (**A** and **B**) Density of SF650-labeled TRPV1 (**A**) and SF650-labeled TRPV4 (**B**) after agonist stimulation. (**C** and **D**) MSD–Δt plots of molecular trajectories under the indicated ligand conditions. SF650-labeled TRPV1 (**E**), SF650-labeled TRPV4 (**F**). Data are means ± SEM of 17–22 cells. *P < 0.05, ***P < 0.001 (one-way ANOVA followed by Dunnett's multiple-comparisons test versus basal level). (**E** and **F**) Correlation between receptor density and time-averaged MSD at each time point for SF650-labeled TRPV1 (**E**) and SF650-labeled TRPV4 (**F**). Each circle represents a time point. Lines are regression lines, and R is the correlation coefficient.

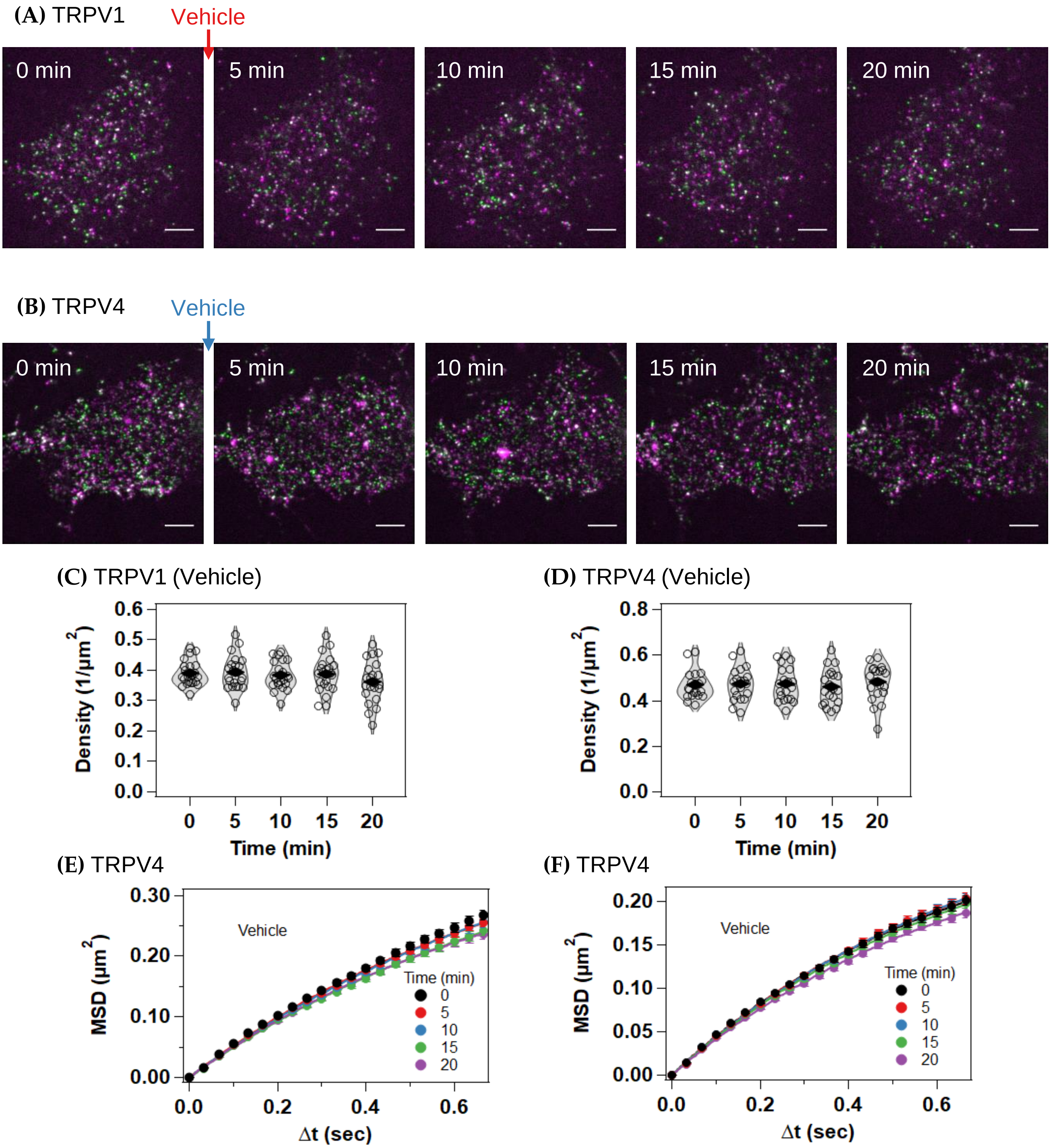

Figure S3. TIRFM images, receptor densities, and MSD–Δt plots of TRPV1 and TRPV4 channels after vehicle stimulation. (**A** and **B**) Representative time-lapse TIRFM images of a TRPV4-KO HEK293 cell expressing JF549 (magenta)- or SF650 (green)-labeled TRPV1 (**A**) or TRPV4 (**B**) after vehicle treatment. Scale bars, 5 μm. (**C** and **D**) Density of JF549-labeled TRPV1 (**C**) and JF549-labeled TRPV4 (**D**). (**E** and **F**) MSD–Δt plots of molecular trajectories after vehicle stimulation. JF549-labeled TRPV1 (**E**) and JF549-labeled TRPV4 (**F**). Data are means ± SEM of 17–22 cells.

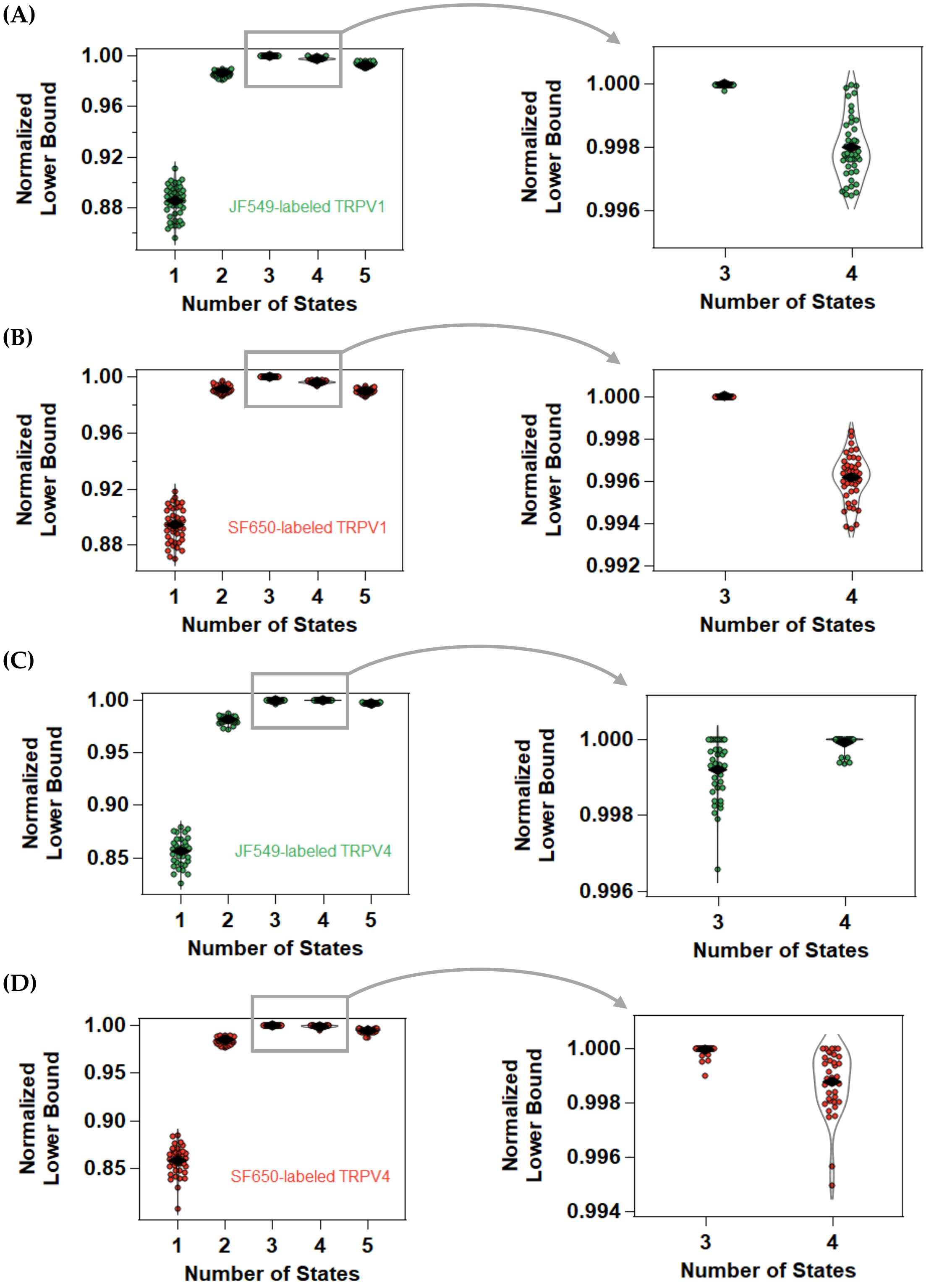

Figure S4. VB-HMM analysis of the TRPV1 and TRPV4 trajectories. (**A**–**D**) Comparison of the normalized likelihood of the clustering results with different numbers of states for JF549-labeled TRPV1 (**A**), SF650-labeled TRPV1 (**B**), JF549-labeled TRPV4 (**C**), and SF650-labeled TRPV4 (**D**). The highest lower bound value corresponds to the number of states with the maxi-mum evidence obtained with the VB-HMM method. Right image shows an enlarged view from three to four states.

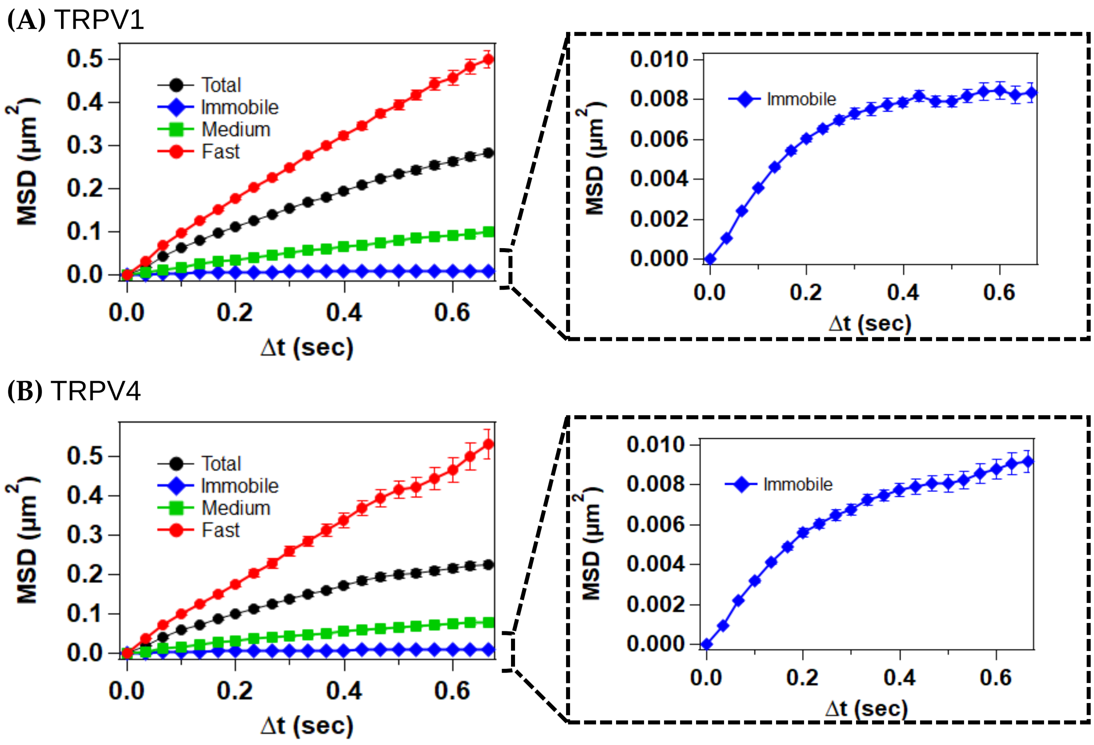

Figure S5. VB-HMM analysis of the TRPV1 and TRPV4 trajectories. (**A** and **B**) MSD–Δt plots of each diffusion state of SF650-labeled TRPV1 (**A**), SF650-labeled TRPV4 (**B**). The immobile, medium, and fast states are shown in blue, green, and red, respectively. Data are means ± SEM of 17–22 cells.

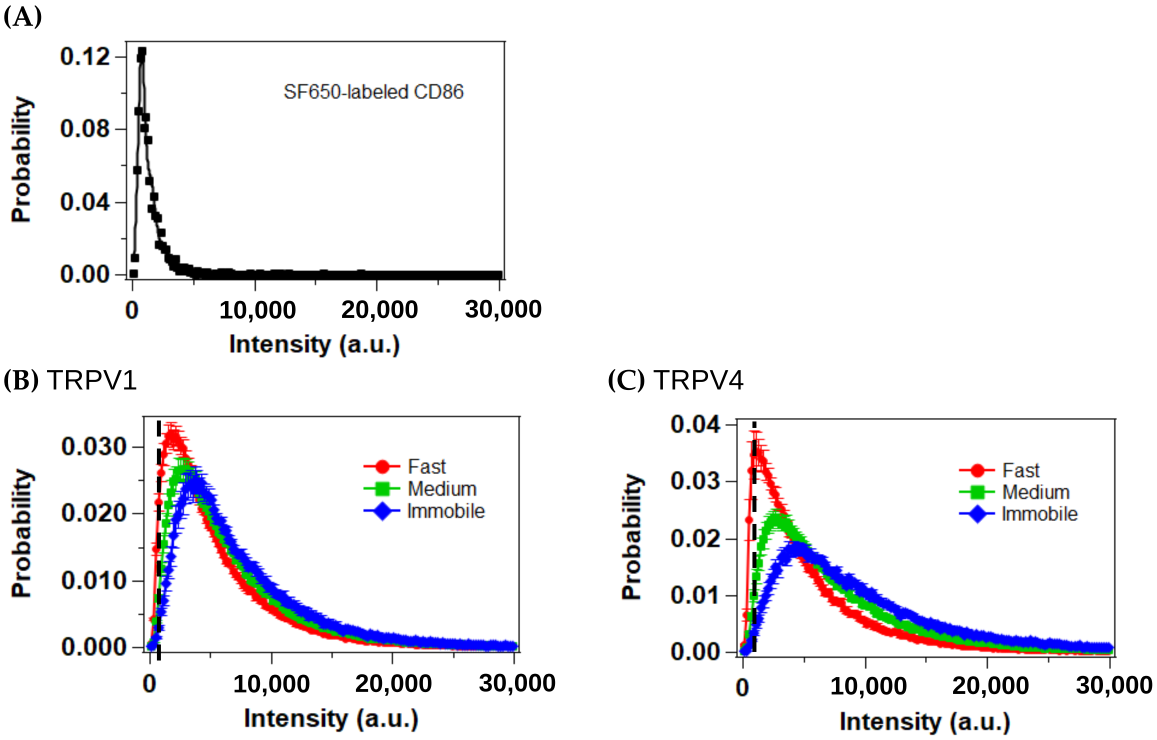

Figure S6. VB-HMM analysis of the oligomeric states of TRPV1 and TRPV4. (**A**) Intensity histograms of SF650-labeled CD86 measured under the same conditions as described for TRPV1 and TRPV4. (**B** and **C**) Histograms of the intensity of SF650-labeled TRPV1 (**C**) and SF650-labeled TRPV4 (**D**) at the basal level. Dashed line represented peak intensity (716 a.u.) of SF650-labeled CD86. The immobile, medium, and fast states are shown in blue, green, and red, respectively.

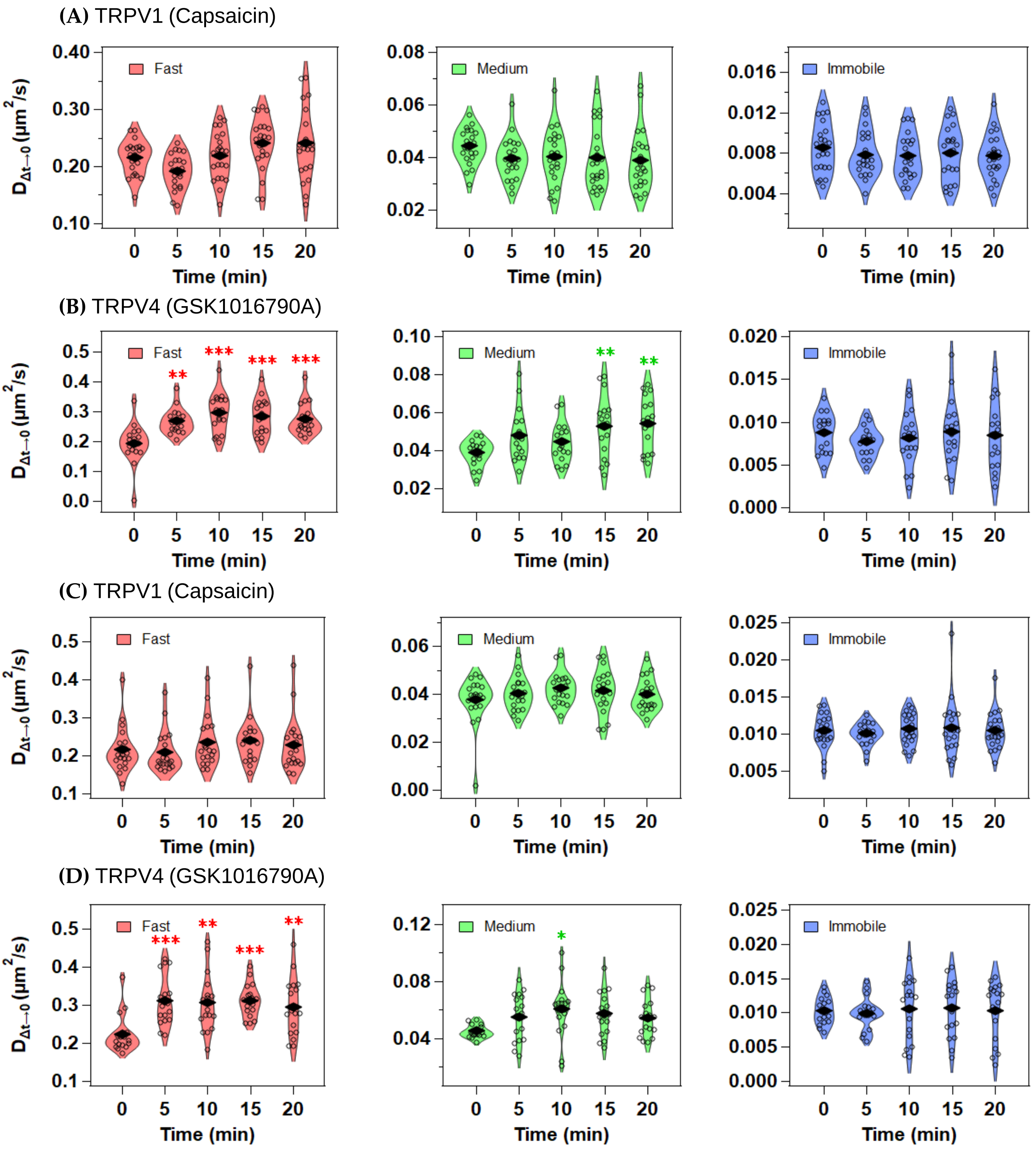

Figure S7. Time-dependent changes in the diffusion coefficient ($D_{\Delta t \to 0}$) of each diffusion state after agonist stimulation. (**A–D**) Violin plots representing the diffusion coefficients of each state estimated from the step size within a frame (30.5 ms) by VB-HMM analysis. Time-dependency of the diffusion coefficients of JF549-labeled TRPV1 (**A**), JF549-labeled TRPV4 (**B**), SF650-labeled TRPV1 (**C**), and SF650-labeled TRPV4 (**D**). The immobile, medium, and fast states are shown in blue, green, and red, respectively. **$P < 0.005$, ***$P < 0.001$ (one-way ANOVA followed by Dunnett's multiple-comparisons test versus basal level).

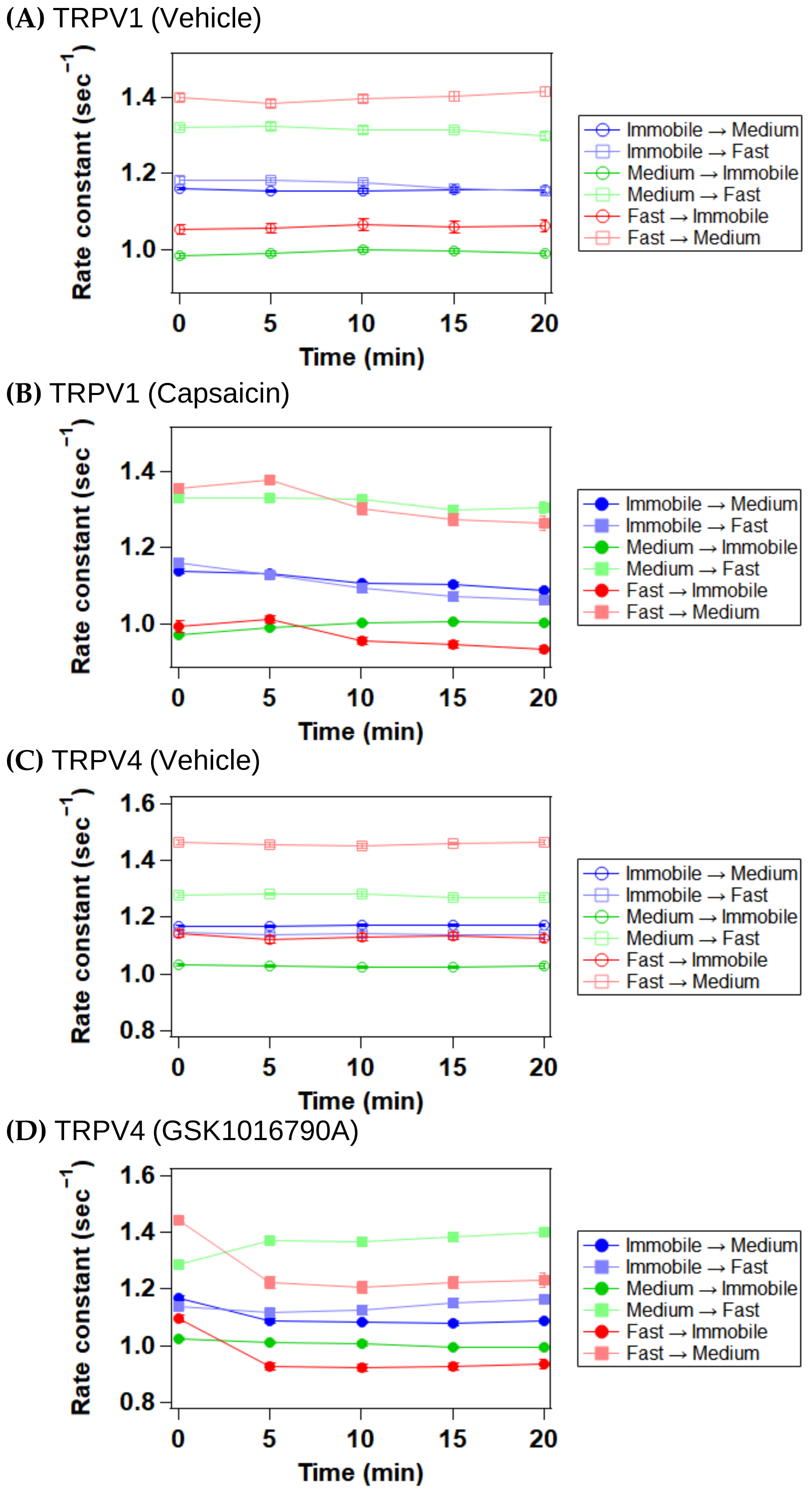

Figure S8. Time-dependent changes in the time constants of the state transition after vehicle or agonist stimulation. (**A**–**D**) Time constants of the state transitions were estimated from the transition probability within a frame (30.5 ms) by VB-HMM analysis. (**A** and **B**) TRPV1 after vehicle (A) or capsaicin stimulation (B). (**C** and **D**) TRPV4 after vehicle (**C**) or GSK1016790A stimulation (**D**). Data are means ± SEM of 17–22 cells.

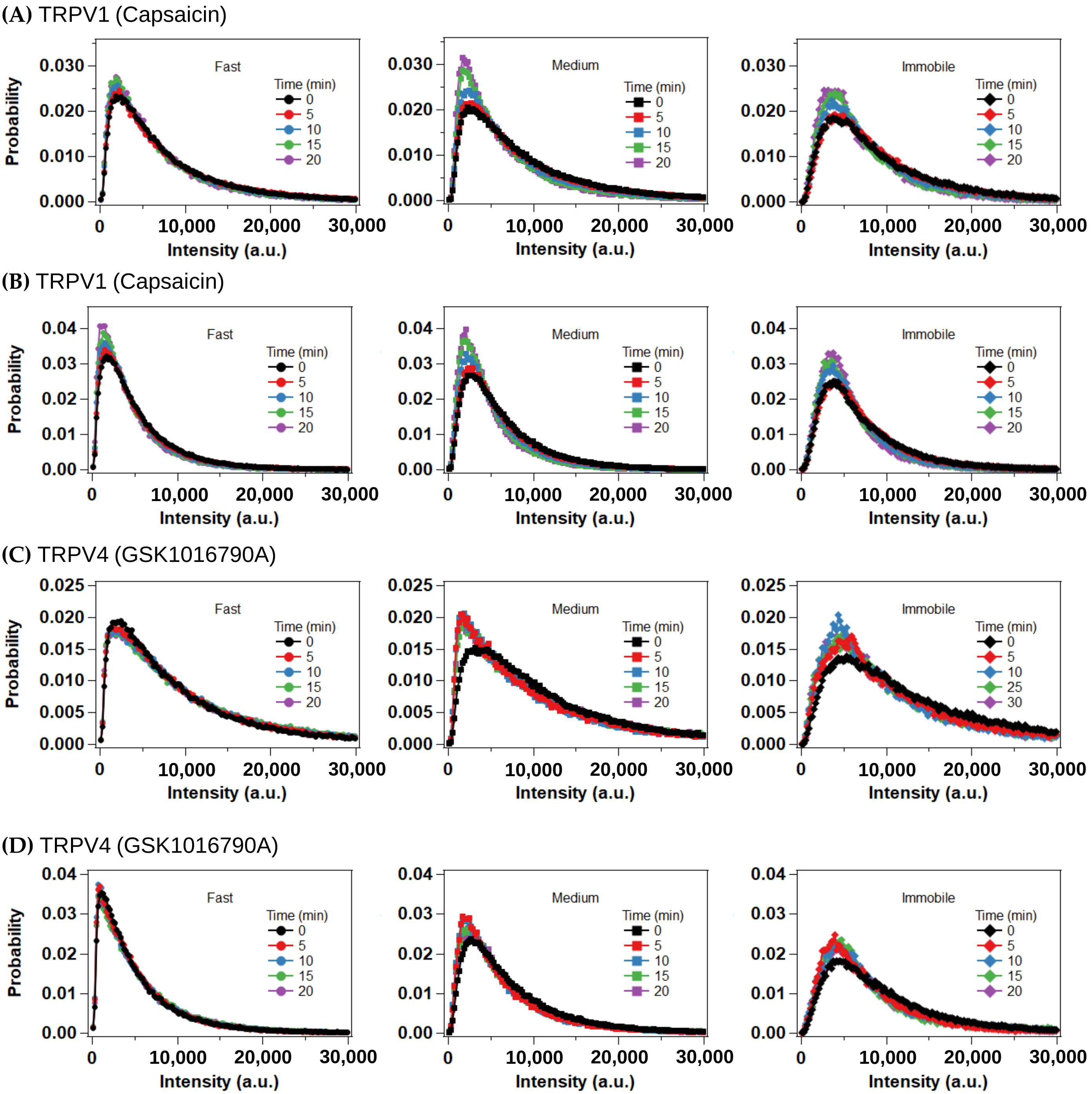

Figure S9. Time-dependent changes in the oligomeric states of TRPV1 and TRPV4 after agonist stimulation. Histogram of the intensity of JF549-labeled TRPV1 (A) and SF650-labeled TRPV1 (B) after capsaicin simulation, and of JF549-labeled TRPV4 (C) and SF650-labeled TRPV4 (D) after GSK1016790A stimulation.

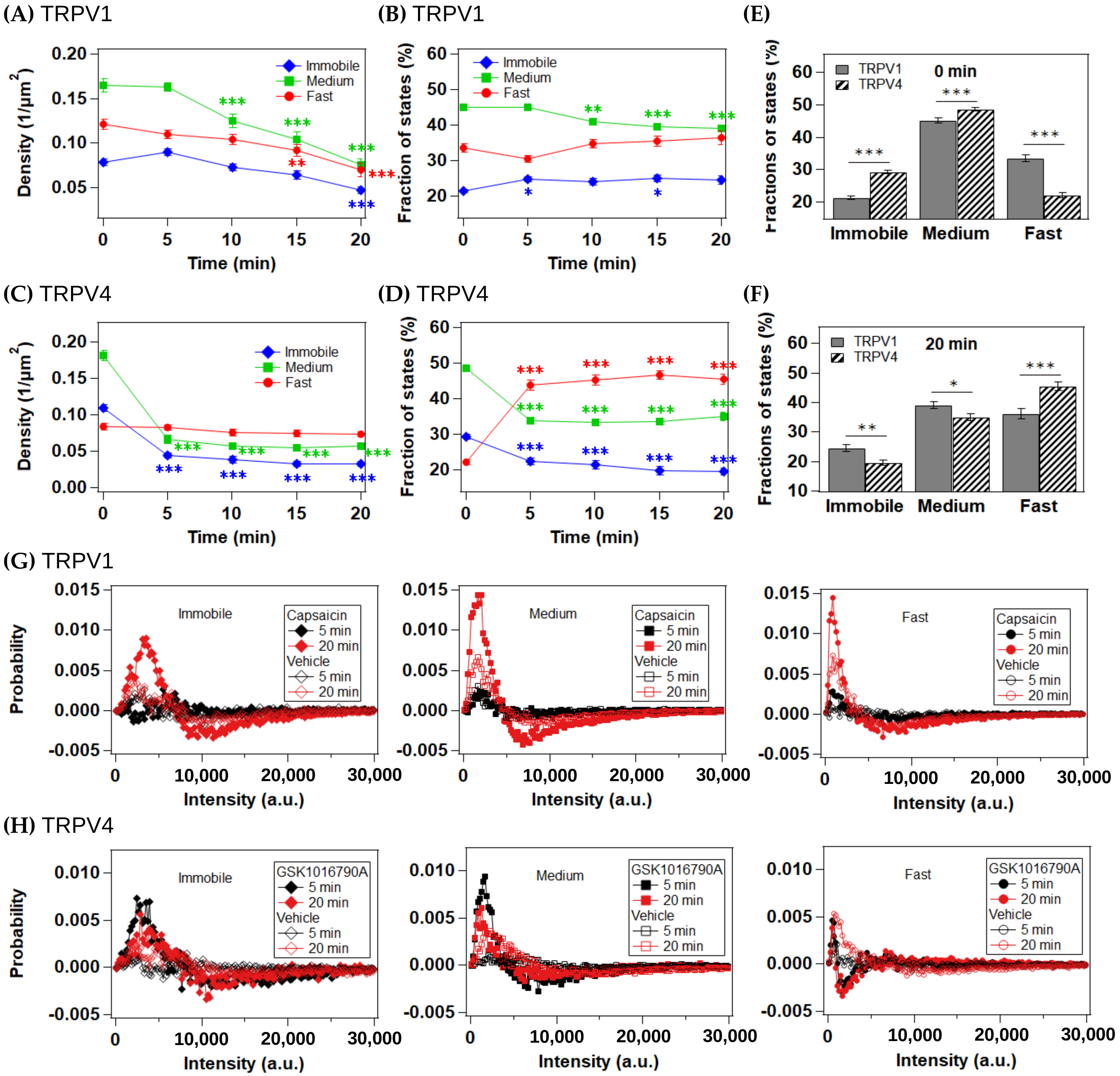

Figure S10. Agonist-induced changes in the dynamics of the TRPV1 and TRPV4 channels. (**A** and **B**) Time-dependent changes in the density of each diffusion state of SF650-labeled TRPV1 upon capsaicin (300 nM) stimulation (**A**), and of TRPV4 upon GSK1016790A stimulation (**B**). (**C** and **D**) Time-dependent changes in the fractions of the diffusion states of SF650-labeled TRPV1 (**C**) and SF560-labeled TRPV4 (**D**) after activation. The immobile, medium, and fast states are shown in blue, green, and red, respectively. Data are means ± SEM of 17-–22 cells. *P < 0.05, **P < 0.005, ***P < 0.001 (one-way ANOVA followed by Dunnett's multiple-comparisons test versus basal level). (**E** and **F**) Comparison of the diffusion state fractions for TRPV1 and TRPV4 at 0 min (**E**) and 20 min (**F**). *P < 0.05; **P < 0.01, ***P < 0.001; two-tailed *t* test when compared with the same diffusion state of TRPV1. (**G** and **H**) Time-dependent changes in the intensity of SF650-labeled TRPV1 (**G**) and SF650-labeled TRPV4 (**H**) after vehicle (black) or agonist stimulation (red).

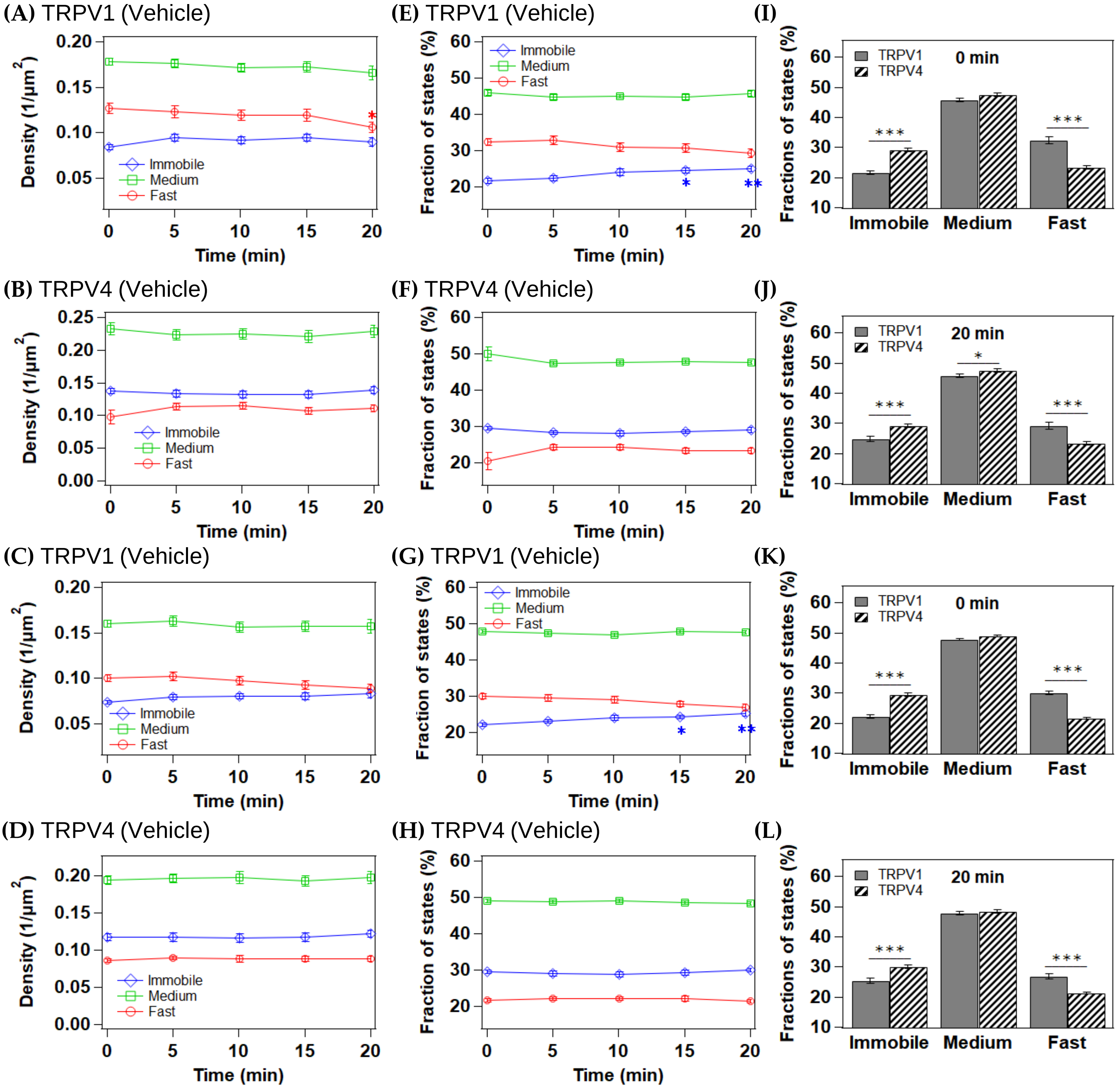

Figure S11. Changes in the dynamics of the TRPV1 and TRPV4 channels after vehicle stimulation. (**A**–**D**) Time-dependent changes in the density of each diffusion state of JF549-labeled TRPV1 (**A**), SF650-labeled TRPV1 (**B**), JF549-labeled TRPV4 (**C**), and SF650-labeled TRPV4 (**D**) after vehicle stimulation. (**E**–**H**) Time-dependent changes in the fractions of the diffusion states of JF549-labeled TRPV1 (**E**), SF650-labeled TRPV1 (**F**), JF549-labeled TRPV4 (**G**), and SF650-labeled TRPV4 (**H**) after vehicle stimulation. The immobile, medium, and fast states are shown in blue, green, and red, respectively. (**I**–**L**) Comparison of the diffusion state fractions of JF549-labeled TRPVs at 0 min (**I**) or 20 min (**J**), and SF650-labeled TRPVs at 0 min (**K**) or 20 min (**L**). *$P < 0.05$; **$P < 0.01$, ***$P < 0.001$; two-tailed *t* test when compared with the same diffusion state of TRPV1.

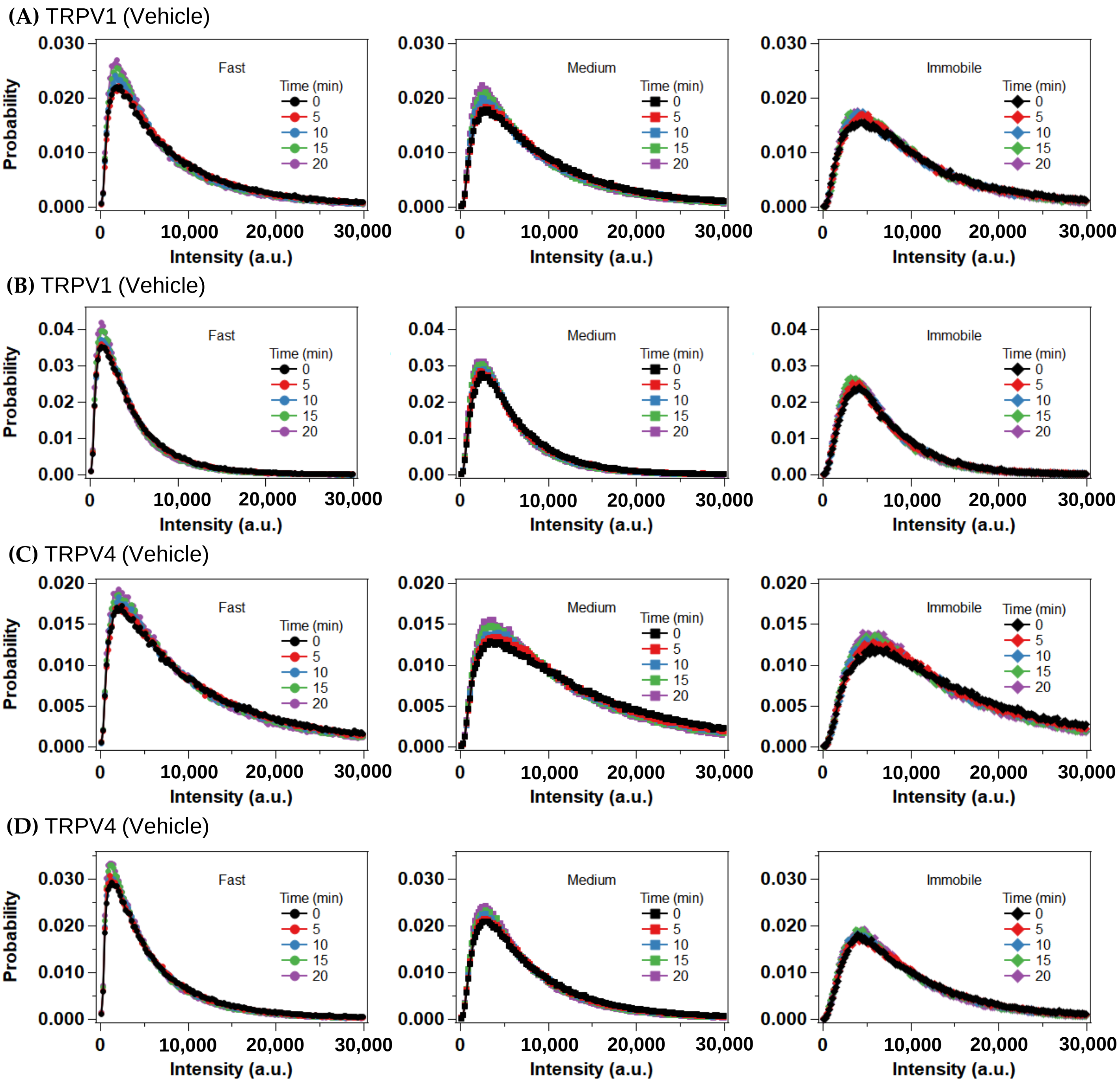

Figure S12. Time-dependent changes in the oligomeric states of TRPV1 and TRPV4 after vehicle stimulation. Histogram of the intensity of JF549-labeled TRPV1 (A), SF650-labeled TRPV1 (B), JF549-labeled TRPV4 (C), and SF650-labeled TRPV4 (D) after vehicle stimulation.

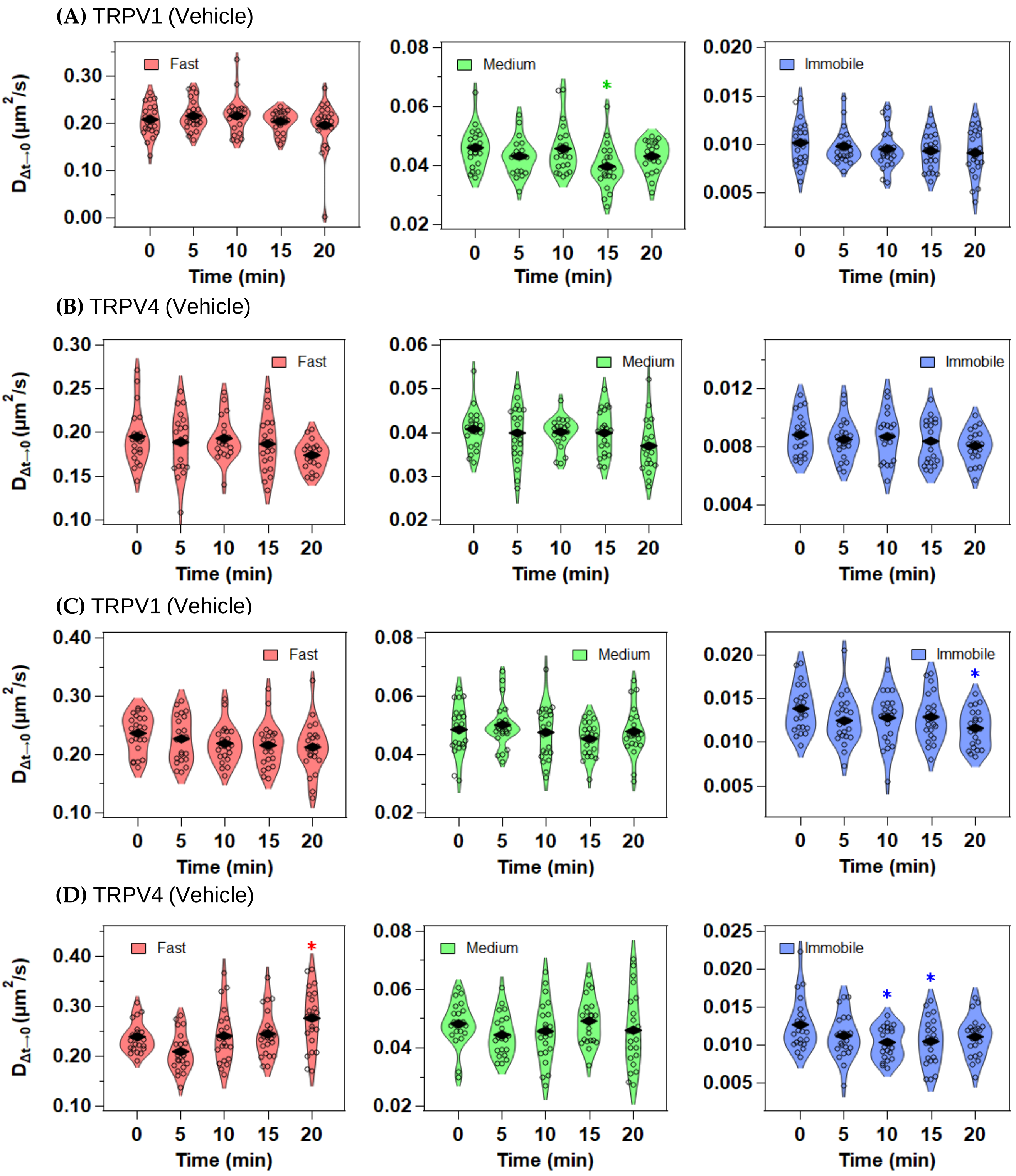

Figure S13. Time-dependent changes in the diffusion coefficient ($D_{\Delta t \to 0}$) of each diffusion state after vehicle stimulation. (**A**–**D**) Violin plots representing the diffusion coefficient of each state estimated from the step size within a frame (30.5 ms) by VB-HMM analysis. Time-dependency of the diffusion coefficients of JF549-labeled TRPV1 (**A**), JF549-labeled TRPV4 (**B**), SF650-labeled TRPV1 (**C**), and SF650-labeled TRPV4 (**D**). The immobile, medium, and fast states are shown in blue, green, and red, respectively. **$P < 0.005$, ***$P < 0.001$ (one-way ANOVA followed by Dunnett's multiple-comparisons test versus basal level).

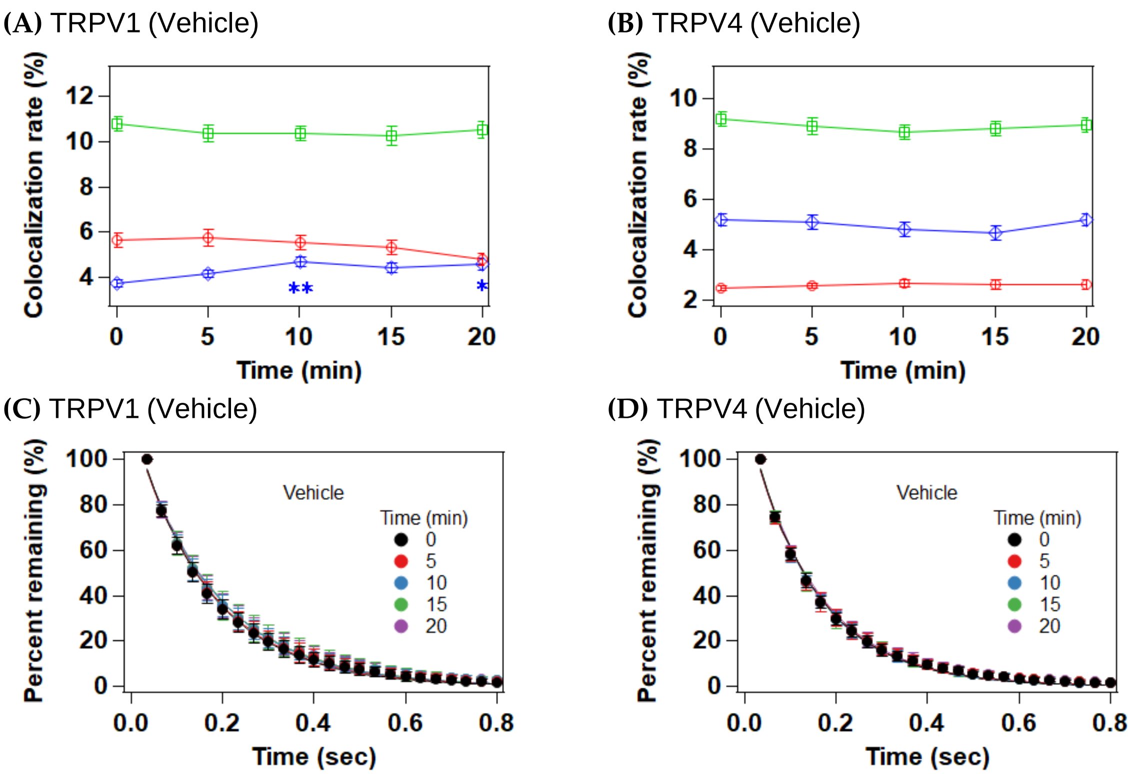

Figure S14. Colocalization analysis of TRPV1 and TRPV4. (**A** and **B**) Time-dependent changes in the colocalized fractions of the diffusion states. TRPV1 after vehicle (**A**), TRPV4 after vehicle (**B**). (**C** and **D**) Time-dependent changes in the percentage remaining of TRPV1 (**C**) and TRPV4 (**D**) after the addition of vehicle. Data are means ± SEM of 17–22 cells. *P < 0.05, **P < 0.005, ***P < 0.001 (one-way ANOVA followed by Dunnett's multiple-comparisons test versus basal level).

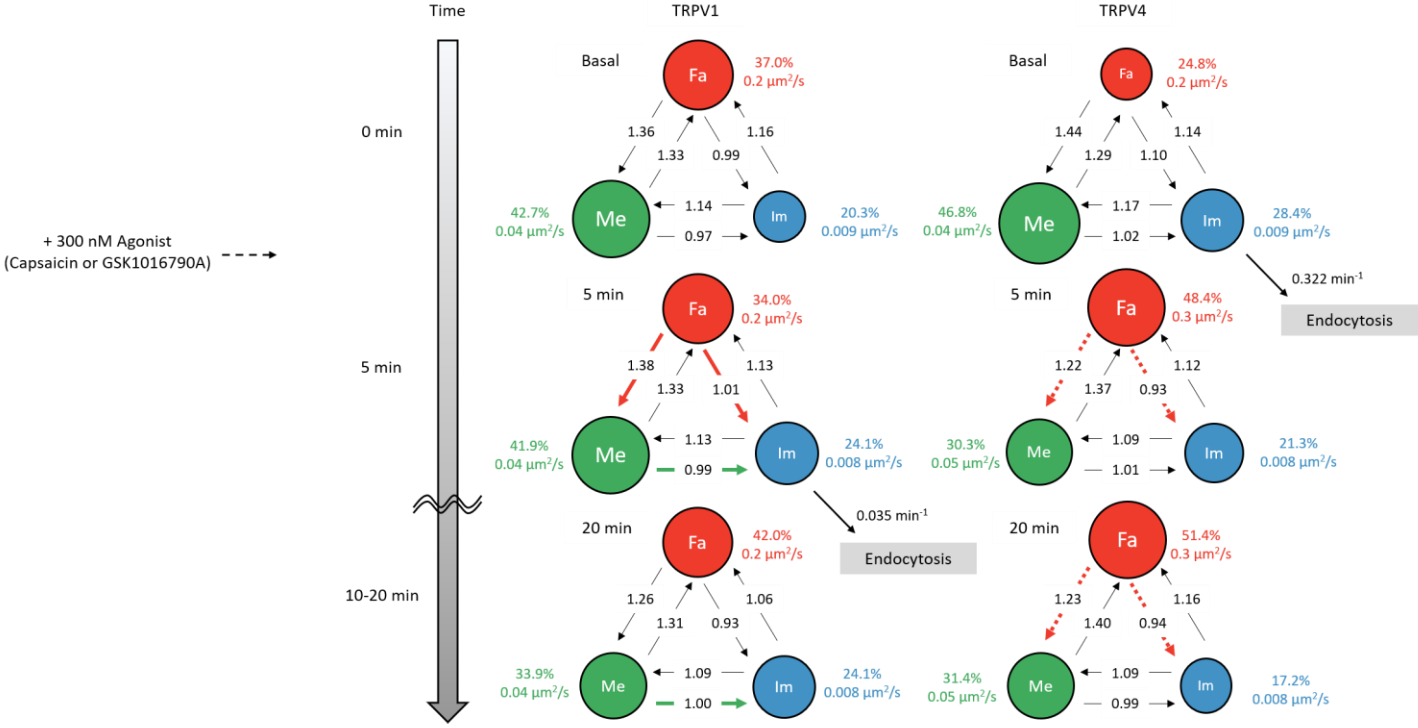

Figure S15. Schematic representation of TRPV1 and TRPV4 channel diffusion in the plasma membrane after activation. The equilibrium among the three diffusion states of TRPV1 or TRPV4 (immobile, medium, and fast) is temporarily altered upon agonist stimulation.
